# Multifunctional redundancy: Impossible or undetected?

**DOI:** 10.1002/ece3.10409

**Published:** 2023-08-16

**Authors:** Bridget E. White, Mark J. Hovenden, Leon A. Barmuta

**Affiliations:** ^1^ School of Natural Sciences University of Tasmania Hobart Tasmania Australia

**Keywords:** ecology, ecosystem functioning, multifunctionality, multifunctional redundancy, redundancy

## Abstract

The diversity‐functioning relationship is a pillar of ecology. Two significant concepts have emerged from this relationship: redundancy, the asymptotic relationship between diversity and functioning, and multifunctionality, a monotonic relationship between diversity and multiple functions occurring simultaneously. However, multifunctional redundancy, an asymptotic relationship between diversity and multiple functions occurring simultaneously, is rarely detected in research. Here we assess whether this lack of detection is due to its true rarity, or due to systematic research error. We discuss how inconsistencies in the use of terms such as ‘function’ lead to mismatched research. We consider the different techniques used to calculate multifunctionality and point out a rarely considered issue: how determining a function's maximum rate affects multifunctionality metrics. Lastly, we critique how a lack of consideration of multitrophic, spatiotemporal, interactions and community assembly processes in designed experiments significantly reduces the likelihood of detecting multifunctional redundancy. Multifunctionality research up to this stage has made significant contributions to our understanding of the diversity‐functioning relationship, and we believe that multifunctional redundancy is detectable with the use of appropriate methodologies.

## Abstract 2

Redundancy, the asymptotic relationship between diversity and ecosystem functioning, and multifunctionality, a monotonic relationship between diversity and multiple functions occurring simultaneously, are significant ecological concepts. However multifunctional redundancy, an asymptotic relationship between diversity and multiple functions occurring simultaneously, is rarely detected. Here we critically review multifunctionality literature to assess if it is truly rare or simply a result of a lack of terminological and methodological consensus.

## 1 INTRODUCTION

Understanding the relationship between community composition and ecosystem function has been a key aim of modern ecology (e.g. Doak et al., 1998; Ehrlich, 1981; Walker, 1992). Experimental studies show that ecosystem productivity increases with increasing species richness and diversity, designated by Tilman (1999) as the diversity‐productivity relationship. The mechanism underlying this relationship is that species differ in their characteristics and thus perform different tasks or, if they perform the same tasks, they do so at different rates. This relationship has been extended to processes beyond productivity, such as nutrient cycling (e.g. Balvanera et al., 2006), with the consensus being that there is an inherent link between ecosystem diversity and function.

Diversity‐functioning relationships are generally considered to be asymptotic, with the rate of the function increasing rapidly as diversity increases initially but then slowing at higher levels of diversity, after which further increases in diversity do not affect the rate of functioning. The asymptotic relationship between diversity and ecosystem functions gives rise to an additional crucial concept: redundancy (Box 1; Fetzer et al., 2015; Galland et al., 2020). If diversity can increase without altering the rate of a particular ecosystem function once a threshold has been reached, then it must also be possible for diversity to decrease without any change in the rate of that function, provided diversity is maintained above a critical level. This implies that highly diverse ecosystems possess some form of redundancy within their communities such that losing one or more species does not alter the rate of a given ecosystem process. Redundancy can be considered through multiple lenses, such as species diversity or functional diversity (Box 1). A trait‐based functional approach has a stronger mechanistic backing (Box 1, Mori et al., 2013) but is more difficult to assess due to higher information requirements.

BOX 1 **Diversity, redundancy and multifunctionality.**

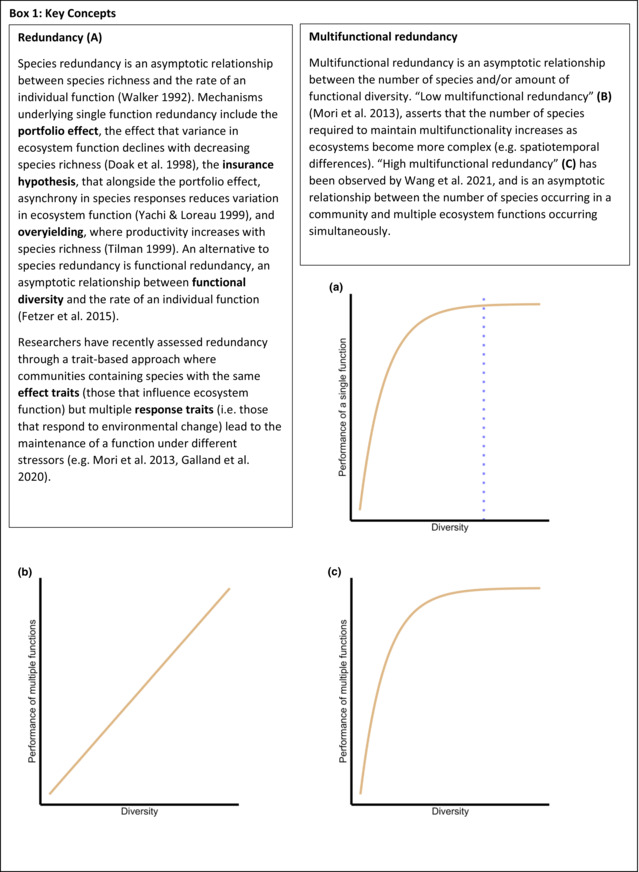

Organisms are multifunctional because their activities influence the rate of several ecosystem functions simultaneously (e.g. plants fix carbon, transpire water and cycle nutrients) Consequently, it is possible that the loss of a particular species might differentially impact the rates of several processes, which undermines the concept of redundancy (Mori et al., 2013). In response, ‘ecosystem multifunctionality’ was coined to describe the relationship between diversity and the simultaneous operation of multiple functions (Hector & Bagchi, 2007), with multiple studies implying a non‐asymptotic relationship between diversity and the number of functions performed in a community (Gamfeldt & Hillebrand, 2008; Mori et al., 2016; Peter et al., 2011). However, it is also possible that multiple functions can be maintained within the ecosystem despite the loss of species: thus, ecosystems could possess multifunctional redundancy. Currently, the field tends towards the thinking that ‘low multifunctional redundancy’ (i.e. no multifunctional redundancy, Box 1, Mori et al., 2013) occurs in ecosystems because the relative contributions of different species to multiple functions changes over time, meaning no species are redundant (see also Hillebrand & Matthiessen, 2009).

Multifunctional redundancy is more challenging to detect than redundancy for a single function. Although redundancy among species performing single functions is often observed (Balvanera et al., 2006), multifunctional redundancy appears rare in both designed experiments and surveys (Li et al., 2021). A meta‐analysis by Hillebrand and Kunze (2020) found that ecosystems typically recover from disturbances, with higher recovery of ecosystem functioning compared to ecosystem composition. They implied that functional redundancy is of key importance here—we argue that because ecosystems are multifunctional, a mechanism underlying recovery could be multifunctional redundancy.

Despite this assertion, we question whether multifunctional redundancy exists or whether methodological problems prevent its detection. We found that the sheer variety in methods and terminology precluded a formal systematic review or meta‐analysis. Instead, we have evaluated two broad fields in multifunctionality research: methods and ecological complexities, and provide insights into how we can move past the current issues in the field.

## 2 HISTORY

### 2.1 Early multifunctionality and multifunctional redundancy

Hector and Bagchi (2007) pioneered multifunctionality research by suggesting that species potentially redundant in the context of a single function may not be redundant when more functions are considered. They found a positive, asymptotic relationship between species richness and the number of functions measured, that is, that species differentially affect processes, and, therefore, multiple species are required to maintain ecosystems. This relationship was discovered in a long‐term experiment consisting of randomly assembled plant communities, and involved multiple community attributes that were not necessarily functions (e.g. above and below ground biomass, nitrogen pools: Spehn et al., 2005). Multifunctionality was subsequently investigated by Gamfeldt et al. (2008) across three communities, finding that functional redundancy was much lower when multiple functions were considered simultaneously compared to single functions. These researchers used different methods of measuring multifunctionality, measured different functions and used functional maxima calculations from monoculture studies (Hillebrand & Matthiessen, 2009), making them difficult to compare despite outward facing similarities. Following from here, multiple researchers failed to detect an asymptotic relationship between multifunctionality and diversity (Maestre et al., 2012; Zavaleta et al., 2010), leading to the concept of low multifunctional redundancy (Box 1). However, these later works are still difficult to compare due to the variety of methods available to calculate multifunctionality.

### 2.2 Calculating multifunctionality

Multifunctionality can be calculated using multiple methods (Byrnes et al., 2014, 2022; Eskelinen et al., 2020), impeding the development of a universally applicable metric. Most of the conceptual development and early empirical studies of multifunctionality retrofitted results from large‐scale, designed experiments such as BIODEPTH (e.g. Gamfeldt & Roger, 2017; Hector & Bagchi, 2007). Several methods were used to retroactively calculate multifunctionality from individually measured functions. Rather than discuss these common methods here, see the comprehensive prior discussion on the four most common ways to measure multifunctionality by Byrnes et al. (2014): the ‘single‐function’, averaging, turnover, and single‐threshold approaches. Instead, we will be describing novel methods of measuring multifunctionality, and how we need to treat our data and methods moving forward in multifunctionality research.

Byrnes et al. (2014) introduced an extension of the threshold approach, which computes the number of functions performing at or above each of multiple thresholds and regressing these values against species richness. Van Der Plas et al. (2016) successfully used this method to quantify multifunctionality in naturally assembled European tree communities. They found a positive relationship between species richness and multifunctionality at low thresholds, peaking when all functions were performing at 37% of their maximum rate, implying multifunctional redundancy was occurring at moderate thresholds. The same analysis, however, also found that the relationship between species richness and multifunctionality became negative when functions were being performed at 76% of their maximum rate. Van Der Plas et al. (2016) suggested that this was true because performing functions at very high rates may compromise the performance of other functions. Overall, using Byrne's novel metric, multifunctional redundancy was detectable at moderate thresholds.

Novel metrics have also been proposed. For example, Eskelinen et al. (2020) developed a metric called ‘slow‐fast multifunctionality’, which assigns positive or negative values to different rates, pools or quality metrics based on whether these metrics are associated with faster or slower cycling rates in plant communities. This metric can be used to assess multifunctionality changes in response to environmental change rather than to changes in species richness. Eskelinen et al. (2020) used the ‘averaging’ method because the threshold approach is not compatible with value assignment. They found complex relationships between watering and fertilizing communities and slow‐fast multifunctionality. Despite the researchers not measuring true ecosystem functions and splitting functions into groups for further analysis ultimately negating the definition of multifunctionality, they provided a novel and interesting method of using multifunctionality techniques.

Dooley et al. (2015) also developed a novel metric based on the Diversity Interaction model (Kirwan et al., 2009) to assess drivers of multifunctionality while incorporating species interactions, functional trade‐offs and the variety of methods used to measure species diversity. This technique incorporates the effect of an individual species on an individual function with the effect of species interaction on said function, and is a matrix equation to incorporate all species across a community. However, their tested system only included four species and the measured ecosystem functions were not ‘true’ ecosystem functions, but instead were biomass and nutrient pools, issues that we discuss in our next section. The flexibility and ability to expand this model is admirable; however, the model became exceptionally complex in a very simple experimental community and may be difficult to apply in more realistic communities.

Byrnes et al. (2022) consolidated previous work, using a method analogous to Hill numbers in diversity metrics. The metric combines two key aspects of multifunctionality: the number of functions being performed and their rate of performance, weighting the two variables against each other. This is similar to the use of Hill numbers to evaluate the relative importance of abundance versus species richness (Hill, 1973). Byrnes et al. (2022) also provided evidence that when ‘q’, their equivalent to a Hill number, equals 0, their metric produces the same value as the ‘averaging’ technique, allowing comparison between prior work and their new metric.

Jing et al. (2020) combined data from major manipulative grassland experiments and forest observational studies to compare across the multiple techniques described by Byrnes et al. (2014). They found that biodiversity–multifunctionality relationships differed depending on the method used, suggesting that some outcomes may be driven more by the method used to analyse data, as opposed to the data itself. This makes it exceptionally difficult for researchers to know if their result is due to an artefact of the method they used, or a true result! Gamfeldt and Roger (2017) emphasised this, noting that seemingly trivial decisions about the choice of technique or threshold impacts research findings.

## 3 MULTIFUNCTIONAL REDUNDANCY: FAILURES TO DETECT

### 3.1 Inconsistent terminology

An ecosystem function is the flow of materials and processing of energy (Naeem, 2008) and is exemplified by photosynthesis. Manning et al. (2018) stipulated that such functions must be measurable as a rate. Nevertheless, ‘Ecosystem function’ is often conflated with ‘ecosystem service’ (such as provision of food and timber, e.g. De Groot et al., 2002), including less tangible benefits (e.g. aesthetics: Kremen, 2005), with many of these services not being measurable as rates. This conflation extends into the multifunctionality literature. Garland et al. (2021) found that 3/82 studies measured ecosystem functions exclusively whereas the vast majority included services such as aesthetics and climate regulation, meaning that biologically irrelevant services (e.g. aesthetics) were being compared with biologically relevant functions (e.g. decomposition), potentially obscuring the biodiversity–multifunctionality relationships. Manning et al. (2018) further maintained that ecosystem multifunctionality studies should only include measurements of ecosystem process rates, or proxies for them, because estimating ecosystem service multifunctionality requires the weighting of functions by human valuation.

The consequences of inflating the number of ‘processes’ by including services are twofold. First, as the number of potential functions and services is infinite, an infinite number of species is required to maximise them, thus preventing the detection of multifunctional redundancy because real species pools are finite. Secondly, ecosystem functions are rate‐limited, whereas qualitatively or subjectively measured services may not have a limit, and such measures are incompatible with any method that requires standardisation with a maximum value (e.g. averaging).

A future terminological issue may be the increasing use of ‘observed taxonomic units’ as a substitute for species in diversity measurements. It is undeniable that response diversity plays a key role in redundancy (Mori et al., 2013), and it is therefore likely to be important in multifunctional redundancy. Multiple microbial studies are moving towards using genomics to measure diversity (e.g. Li et al., 2021; Mori et al., 2016), while splitting groups of taxa into coarse functional roles. Even though this work provides useful information about the effects of species diversity on functioning, it does not allow us to explore mechanisms underlying redundancy.

### 3.2 Problems with defining maxima

Averaging, threshold, multiple threshold and Hill number‐based methods of calculating multifunctionality all require measuring a maximum level of functioning. Overestimating maxima would bias against detecting redundancy (Box 2) and studies differ in how maxima are measured. For instance, several studies use a single observation from an experimental community (e.g. Jing et al., 2020; Pasari et al., 2013; Wang et al., 2022) or from monocultures (e.g. Gamfeldt & Roger, 2017; Hector & Bagchi, 2007 who used maxima from the BIODEPTH experiment; Spehn et al., 2005). Still others are ambiguous about their methods (Christianen et al., 2023; Moi et al., 2021). To progress, Byrnes et al. (2014) suggested that maximum functioning should be measured as an average of the highest performing communities (e.g. Anujan et al., 2021; Lefcheck et al., 2015; Mori et al., 2016; Van Der Plas et al., 2016; Zavaleta et al., 2010) to prevent unusual outliers from skewing measures of multifunctionality. However, the methods used to select maximum functioning rates remains inconsistent across the literature.

BOX 2 **The issue with incorrectly calculating maximum rate.**

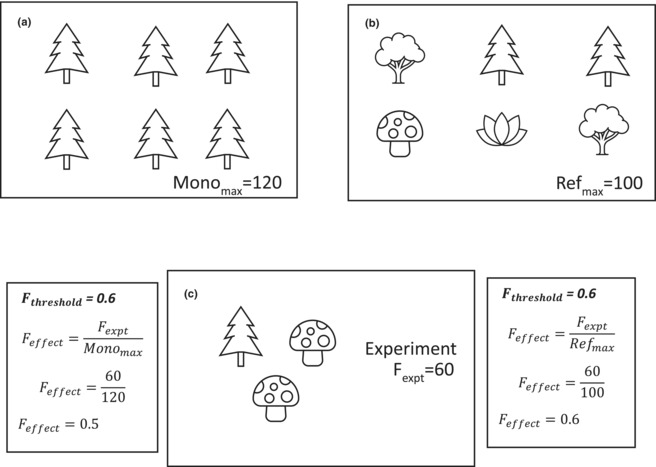

Assume a function in a reference ecosystem has a value of 100 units (a). However, functioning was measured in a monoculture where the monoculture maximum was measured to be 120 units (b). We perform an experiment to alter our community and measure functioning to be 60 units (c). If we standardise that function according to the maximum in monoculture, it would be occurring at 0.5 units (bottom left). However, if we standardise the function according to the reference ecosystem, it occurs at 0.6 units. If a functional threshold is set at 0.6, and we take the monoculture max, the functional threshold would not be met (bottom left). However, if we used the reference maximum, the threshold would be met (bottom right). If the threshold is not met, it would be impossible to detect redundancy for that function, and therefore multifunctional redundancy.

Species interactions or other assembly processes can affect the performance of a function in a natural community and thus enhance or limit the functional maxima which means that transferring maxima measured elsewhere can be flawed. Negative biotic interactions, for example, competition, may reduce a reference functional maximum in a natural community compared to a researcher‐assembled community. Positive biotic interactions may increase a reference functional maximum through facilitation compared to a researcher‐assembled community. Every community will have a slightly different reference maximum, thus designing experiments around the concept of a functional reference maximum will assist the production of accurate estimates of multifunctionality. This will likely involve taking a multifunctionality measurement, manipulating the community somehow, and retaking the measurement to see if multifunctionality was altered in order to ensure that relative functional maxima are compared between different communities (Box 2).

### 3.3 Measuring biomass versus rates

Many studies use biomass as a proxy for rate‐based measurements of functions (e.g. Gamfeldt & Hillebrand, 2008; Moi et al., 2021), which may obscure redundancy relationships. For example, aquatic food webs often have inverted pyramids of biomass due to the shorter generation times of plankton compared to their consumers (Mccauley et al., 2018). This means that biomass pools cannot be maximised simultaneously. If such biomass pools are used as proxies for rate, multifunctional redundancy cannot be achieved as there is no ability for multiple biomass pools to asymptote simultaneously. Recasting such food webs in terms of production re‐establishes the pyramidal shape (Mccauley et al., 2018). In order to use biomass as a proxy for a function, correlations between biomass and a rate must be established (e.g. Garland et al., 2021) otherwise functioning cannot be maximised and incorrect values are used in analyses, (e.g. Box 2), potentially altering the observed relationship between diversity and multifunctionality.

### 3.4 Correlated functions

Multifunctional redundancy may be obscured or exaggerated if functions included in a metric are correlated. However, significant complexity underlies this: how are functions correlated? First, we split the relationship that functions have with each other into two categories: ‘dependent functions’ and ‘parallel functions’.

Dependent functions are those that are directly contingent on another function. For example, gross primary productivity (carbon sequestration) and grazing (movement of carbon up the food chain) are positively correlated because grazing can *only* increase if GPP increases. This type of correlation is solved by measuring rates instead of biomass, because rates can be simultaneously maximised whereas biomass pools cannot be maximised.

Parallel functions are those that are driven by independent processes. However, parallel functions can have the same response. For example, transpiration and gross primary productivity are independent processes, but both respond to light and water availability (Jones, 2013). If parallel functions are positively correlated and contained within a single species, multifunctional redundancy will be lower, because adding or removing such a species results respectively increases or decreases multifunctionality. By contrast, if the parallel functions are negatively correlated and contained within the same species, such as wood rotting processes where carbon sequestration declines as decomposition increases, multifunctional redundancy could be neutrally affected. The rates of parallel functions may also change based on environmental changes, for example, Küpper et al. (2008) found that when iron is limited in a marine cyanobacterium, nitrogen fixation is reduced but photosynthetic rates are maintained, which both means that not only species loss, but physiological changes in individuals are important, leading to complex relationships.

Furthermore, if parallel correlated functions occur in different species (e.g. nitrogen‐fixing bacteria and a photosynthetic plant), these relationships may be affected by the presence or absence of other species in the system, their abilities to interact with other species and whether the correlated functions are overall positive or negative.

Using species diversity techniques, Byrnes et al. (2022) attempted a work around for correlated functions, suggesting that calculating the ‘overlap’ in different functions and removing this to calculate an effective number of functions, equivalent to effective number of species, is a work around for managing correlated functions.

Overall, rather than attempting to incorporate these complexities into experiments, the way we perform experiments to determine multifunctionality could assist in incorporating these complexities while reducing our need to directly consider them while selecting functions to measure. It is worth noting that negative correlations within a single species are rare, and therefore may not affect multifunctionality metrics. Calculating metrics from reference maxima will also allow us to incorporate these correlated functions, without having to fully understand them.

### 3.5 Ecological complexities

#### 3.5.1 Multitrophic complexities

Multifunctionality studies often occur within a single trophic level (e.g. Hector & Bagchi, 2007), thus simplifying ecosystem complexities. Lefcheck et al. (2015) concluded that confining multifunctionality test to a single trophic level may underestimate biodiversity effects on multifunctionality. They substantiated this by studying multitrophic multifunctionality in many one‐ to three‐species systems, and found that the effect of biodiversity on multifunctionality increased both when more functions were considered and when the effect of diversity on multifunctionality was considered at higher trophic levels over lower ones.

Soliveres et al. (2016) analysed data from 150 grasslands, across multiple trophic levels from soil microbes through to grazers and predators, finding that multifunctionality was highest when multiple trophic groups were considered over a single functional group. They studied ecosystem *services* over ecosystem functions in their calculation of the multifunctionality metric, and used multiple single‐threshold tests of multifunctionality in their analysis. This consideration of multiple trophic levels is important in multifunctionality research; however, issues with the type of functions being measured may be preventing the detection of high multifunctional redundancy.

#### 3.5.2 Spatiotemporal complexities

It is possible for species to coexist in space but not in time or vice versa. Accordingly, there are trade‐offs between different functions, meaning that relationships between biodiversity and multifunctionality are highly complex (Hillebrand & Matthiessen, 2009). Mori et al. (2018) pointed out that all such studies thus far have considered α‐diversity loss, but loss of β‐diversity is also important due to spatial complexity because we cannot expect all functions to reach their maxima in one location due to functional trade‐offs. Many multifunctionality studies only consider one level of spatial and temporal complexity (e.g. Bradford et al., 2014; Gamfeldt et al., 2008; Hector & Bagchi, 2007; Peter et al., 2011), and do not consider this spatial turnover. Using different vegetation layers, Wang et al. (2021) compared multifunctionality over different spatial scales, finding that multifunctionality varied spatially, meaning that spatiotemporal complexity is important to consider in multifunctionality studies. Furthermore, asynchronous biomass production of co‐occurring grassland species led to a high degree of temporal stability in productivity at the community level (Ma et al., 2017), demonstrating how important it is to consider whole ecosystems over space and time when calculating multifunctionality.

#### 3.5.3 Community assembly processes, interactions and redundancy

Community assembly processes develop key biotic and abiotic interactions underlying redundancy. As early as 1980, Yodzis (1980) found that deterministic community assembly processes are important for developing paired species interactions. This was exemplified in a study of the effects of random versus non‐random extinctions of marine invertebrates on bioturbation which found that non‐random extinctions reduced bioturbation more than random extinctions (Solan, 2004). In the 1990s, as redundancy was being explored, species linkages and interactions were identified as key drivers underlying redundancy (Doak et al., 1998; Johnson et al., 1996; Lawton & Brown, 1994; Yachi & Loreau, 1999). Despite not explicitly using the term ‘redundancy’ and using an artificially created community, Downing et al. (2014) used a zooplankton experiment to find that weak interactions reduced population variability in ‘variable’ environments, implying that weak interactions may have a key role in redundancy.

Despite these clear connections between community assembly, species interactions and redundancy, community assembly processes are often ignored in multifunctionality research. For example, the BIODEPTH project design, which has been used by later studies of multifunctionality (e.g. Gamfeldt & Roger, 2017) randomly selected species from different functional groups to produce experimental communities of different biological and functional diversities (Spehn et al., 2005). Furthermore, Slade et al. (2017) studied the relationship between diversity, interspecies interactions and multifunctionality in dung beetle communities and found that species interactions can increase or decrease functions. Meyer et al. (2018) found that multifunctionality increased with biodiversity but was limited by negative correlations between functions. Multifunctionality was also more limited when the environment was altered rather than when species composition was altered. The role of community assembly, taxonomic composition and functional trait composition on multifunctionality was reviewed by Butterfield et al. (2016). They used community average trait values, capturing the idea that the most dominant species in a system contributes the most to a trait class (e.g. body size) and drives the average quantitative value of that trait. Their synthesis concluded that community assembly determines whether community average trait values or functional diversity drive multifunctionality processes.

Overall, in multifunctionality research, the dearth of communities developed by community assembly processes could significantly hamper our ability to detect multifunctional redundancy. Designing diversity–multifunctionality experiments that incorporate community assembly processes will be key to increasing the possibility of detecting multifunctional redundancy, as well as giving us a more realistic understanding of multifunctionality.

## 4 DETECTING MULTIFUNCTIONAL REDUNDANCY: EXEMPLAR

Despite the foregoing issues, recent examples demonstrate the feasibility of detecting high multifunctional redundancy. Li et al. (2021) sampled two microbial soil communities that varied naturally in diversity and progressively diluted them with sterile soil. This selectively removed certain species, confirming that species loss was non‐random. They left these samples for 60 days to ensure overall community abundance was the same despite variations in diversity. The functions they selected were measurable as rates (respiration, nitrogen fixation, nitrification and breakdown of organic material) and they calculated multifunctionality using the averaging technique. Overall, they found that species loss reduced multifunctionality more in the lower diversity site than in the higher diversity site, implying that high multifunctional redundancy occurred when soil diversity was higher. They then compared functional redundancy between sites, finding that the higher diversity soil had more taxa attributed to each functional group compared to the lower diversity soil, suggesting that functional group redundancy led to these results. Furthermore, when they analysed each function individually, they found that functions with the highest diversity among contributors were the most likely to show redundancy, whereas functions that relied on a smaller number of highly specialised taxa had significantly lower process rates, especially in the lower diversity soil. This study implies that when natural communities are manipulated with true ecosystem functions measured, high multifunctional redundancy *is* possible to detect, and, as we refine our methods further, we should be able to find other instances of high multifunctional redundancy.

## 5 CONCLUSIONS

### 5.1 Consequences of using inappropriate methods

Clearly, using inappropriate methods to examine the relationship between diversity and multifunctionality may lead to artefacts. Often, researchers studying the effects of biodiversity on functions randomly delete species and compare the resulting ecosystem function with its prior value or with that from a monoculture treatment. For example, Gamfeldt and Roger (2017), Hector and Bagchi (2007) and Jing et al. (2020) used BIODEPTH data to study multifunctionality, which used randomly selected subsets of species from functional groups to generate communities (Spehn et al., 2005). This approach would underestimate any relationships between diversity and functioning as they would be more likely to be an artefact of the experimental design, rather than of ecological mechanisms. Species losses from real perturbations are unlikely to be random. Environmental changes often selectively remove a suite of species that share certain response traits. For example, Solan (2004) found that random removal of marine invertebrates resulted in slower loss of a function (bioturbation) than if species were removed non‐randomly, and Larsen et al. (2005) found that larger bodied bumble bees and dung beetles were both more extinction prone and more functionally efficient, meaning that species loss was non‐random and resulted in greater loss of functional stability. Furthermore, when these response traits are correlated with effect traits, function loss occurs faster than when traits are uncorrelated (Solan, 2004) leading to lower functional redundancy. The observation that non‐random species loss affects functioning more than random species loss has implications for many multifunctionality studies.

### 5.2 How to progress

In order to progress multifunctionality research, there are key areas that need to be addressed. Here we outline each of these areas in turn.

#### 5.2.1 Definitions

To improve the chance of detecting high multifunctional redundancy, researchers *must* be explicit in their choices of rate‐based ecosystem functions. Including a large number of other ecosystem services or non‐rate‐related resource pools in measures of multifunctionality may hide underlying mechanisms under unnecessary, costly complications. If ecosystem services need to be quantified, then a separate multiservice measurement needs to be included as a separate step (e.g. Manning et al., 2018). However, for better understanding of purely ecological phenomena, rate‐based multifunctionality metrics are key.

#### 5.2.2 Calculations and data management

Multifunctionality metrics will continue to be developed and applied in novel ways. Researchers must be flexible: open access data that are presented in a simple manner will allow for new findings, or confirm what work has already been completed. Models that can incorporate ecological complexities such as Dooley et al. (2015) will likely be important in the future. Clearly stating how each function is measured and comparing each function to a *reference* maximum, will allow for the appropriate use of current and upcoming methods. The methods that researchers use to calculate multifunctionality must also be presented explicitly. Finally, as we currently are still finding the most appropriate methods to measure multifunctionality, researchers should be using and clearly explaining their multiple methods of measuring multifunctionality.

#### 5.2.3 Experiments on natural versus constructed communities

Manipulating naturally assembled communities instead of constructing them is required for truly measuring multifunctionality. Constructed communities are ideal for measuring individual functions (Marquard, Weigelt, Temperton, et al., 2009), and have led to significant development in the biodiversity‐functioning space (Allan et al., 2011; Marquard, Weigelt, Roscher, et al., 2009; Roscher et al., 2012). However, as we move forward in the investigations of biodiversity‐functioning relationships, we must acknowledge that we do not fully understand the complexities of natural ecosystems from their formation through to current drivers of stability. We can still perform tests and studies to understand underlying mechanisms, but these tests must begin with a full, natural ecosystem that we can manipulate to remove species or change abiotic conditions. Repurposing experiments originally designed for measuring single functions may hide multifunctional redundancy. Experiments in natural systems incorporate complexities (e.g. soil microbial systems) and the weak and the strong biotic and abiotic interactions that are key for redundancy relationships. The outcomes of these studies will undoubtedly contain unexplained variation due to unknown mechanisms, but will allow us to focus on mechanisms underlying these responses, and better understand natural systems (Box 3; Pinheiro et al., 2022).

BOX 3 **The continuum of biodiversity‐functioning studies.**

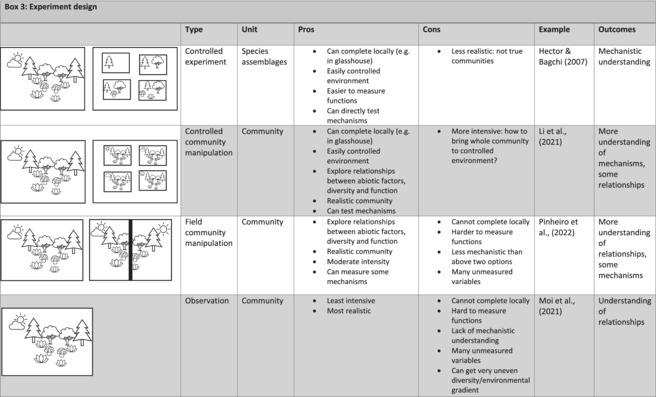

Biodiversity‐functioning studies can range from experimenting on structured species assemblages (top row) through to observing communities without manipulations (fourth row). Studies can be considered in vitro (top two rows), where species or whole communities are manipulated in a laboratory, glasshouse or mesocosm, or in vivo (bottom two rows), where communities are either manipulated or observed in the field. Experimental manipulations are key for exploring mechanisms underlying biodiversity‐functioning relationships, and manipulations on communities that have experienced community assembly processes will give us a greater understanding of how functioning responds to biodiversity alterations.

## 6 CONCLUSION

Multifunctionality research up to this point has allowed us to expand our understanding of the complexity of biodiversity–ecosystem functioning relationships. However, to untangle it, further we must tread carefully through the web of existing methodology to design appropriate studies. Multifunctional redundancy is intuitively appealing but has been exceptionally difficult to detect given the rigidity and complexity of work required to achieve this. Nevertheless, understanding multifunctional redundancy is crucial for ecosystem management, particularly because it allows us to continue answering the question: why is diversity important? Moreover, it gives us meaningful insights into questions such as: how much diversity can we afford to lose?

## AUTHOR CONTRIBUTIONS

**Bridget E. White:** Conceptualization (lead); writing – original draft (lead); writing – review and editing (equal). **Mark J. Hovenden:** Supervision (supporting); writing – original draft (supporting); writing – review and editing (equal). **Leon A. Barmuta:** Conceptualization (supporting); funding acquisition (lead); supervision (lead); writing – original draft (supporting); writing – review and editing (equal).

## FUNDING INFORMATION

This research was supported by the Australian Research Council Discovery Program (DP190102837) to Barmuta, Robson and Death, and Bridget White has additional scholarship support from the University of Tasmania Research Training Program.

## CONFLICT OF INTEREST STATEMENT

The authors declare no competing interests.

## Data Availability

Data sharing is not applicable to this article as no new data were created or analysed in this study.

## References

[ece310409-bib-0001] Allan, E. , Weisser, W. , Weigelt, A. , Roscher, C. , Fischer, M. , & Hillebrand, H. (2011). More diverse plant communities have higher functioning over time due to turnover in complementary dominant species. Proceedings of the National Academy of Sciences of the United States of America, 108, 17034–17039. 10.1073/pnas.1104015108 21949392 PMC3193239

[ece310409-bib-0002] Anujan, K. , Heilpern, S. A. , Prager, C. M. , Weeks, B. C. , & Naeem, S. (2021). Trophic complexity alters the diversity–multifunctionality relationship in experimental grassland mesocosms. Ecology and Evolution, 11, 6471–6479. 10.1002/ece3.7498 34141232 PMC8207441

[ece310409-bib-0003] Balvanera, P. , Pfisterer, A. B. , Buchmann, N. , He, J.‐S. , Nakashizuka, T. , Raffaelli, D. , & Schmid, B. (2006). Quantifying the evidence for biodiversity effects on ecosystem functioning and services. Ecology Letters, 9, 1146–1156. 10.1111/j.1461-0248.2006.00963.x 16972878

[ece310409-bib-0004] Bradford, M. A. , Wood, S. A. , Bardgett, R. D. , Black, H. I. J. , Bonkowski, M. , Eggers, T. , Grayston, S. J. , Kandeler, E. , Manning, P. , Setälä, H. , & Jones, T. H. (2014). Discontinuity in the responses of ecosystem processes and multifunctionality to altered soil community composition. Proceedings of the National Academy of Sciences of the United States of America, 111, 14478–14483. 10.1073/pnas.1413707111 25246582 PMC4210050

[ece310409-bib-0005] Butterfield, B. J. , Camhi, A. L. , Rubin, R. L. , & Schwalm, C. R. (2016). Tradeoffs and compatibilities among ecosystem services. In G. Woodward & D. A. Bohan (Eds.), Ecosystem services: From biodiversity to society, part 2 (pp. 207–243). Elsevier. 10.1016/bs.aecr.2015.09.002

[ece310409-bib-0006] Byrnes, J. E. K. , Gamfeldt, L. , Isbell, F. , Lefcheck, J. S. , Griffin, J. N. , Hector, A. , Cardinale, B. J. , Hooper, D. U. , Dee, L. E. , & Emmett Duffy, J. (2014). Investigating the relationship between biodiversity and ecosystem multifunctionality: Challenges and solutions. Methods in Ecology and Evolution, 5, 111–124. 10.1111/2041-210x.12143

[ece310409-bib-0007] Byrnes, J. E. K. , Roger, F. , & Bagchi, R. (2022). Understandable multifunctionality measures using Hill numbers. Oikos, 2023, e09402. 10.1111/oik.09402

[ece310409-bib-0008] Christianen, M. J. A. , Smulders, F. O. H. , Vonk, J. A. , Becking, L. E. , Bouma, T. J. , Engel, S. M. , James, R. K. , Nava, M. I. , De Smit, J. C. , Van Der Zee, J. P. , Palsbøll, P. J. , & Bakker, E. S. (2023). Seagrass ecosystem multifunctionality under the rise of a flagship marine megaherbivore. Global Change Biology, 29, 215–230. 10.1111/gcb.16464 36330798 PMC10099877

[ece310409-bib-0009] De Groot, R. S. , Wilson, M. A. , & Boumans, R. M. J. (2002). A typology for the classification, description and valuation of ecosystem functions, goods and services. Ecological Economics, 41, 393–408. 10.1016/s0921-8009(02)00089-7

[ece310409-bib-0010] Doak, D. F. , Bigger, D. , Harding, E. K. , Marvier, M. A. , O'Malley, R. E. , & Thomson, D. (1998). The statistical inevitability of stability‐diversity relationships in community ecology. The American Naturalist, 151, 264–276. 10.1086/286117 18811357

[ece310409-bib-0011] Dooley, Á. , Isbell, F. , Kirwan, L. , Connolly, J. , Finn, J. A. , & Brophy, C. (2015). Testing the effects of diversity on ecosystem multifunctionality using a multivariate model. Ecology Letters, 18, 1242–1251. 10.1111/ele.12504

[ece310409-bib-0012] Downing, A. L. , Brown, B. L. , & Leibold, M. A. (2014). Multiple diversity‐stability mechanisms enhance population and community stability in aquatic food webs. Ecology, 95, 173–184. 10.1890/12-1406.1 24649657

[ece310409-bib-0013] Ehrlich, P. E. (1981). Extinction: The causes and consequences of the disappearance of species . [WWW Document]. eweb:35542. Retrieved March 6, 2021, from https://repository.library.georgetown.edu/handle/10822/788604

[ece310409-bib-0014] Eskelinen, A. , Gravuer, K. , Harpole, W. S. , Harrison, S. , Virtanen, R. , & Hautier, Y. (2020). Resource‐enhancing global changes drive a whole‐ecosystem shift to faster cycling but decrease diversity. Ecology, 101, e03178. 10.1002/ecy.3178 32870523

[ece310409-bib-0015] Fetzer, I. , Johst, K. , Schäwe, R. , Banitz, T. , Harms, H. , & Chatzinotas, A. (2015). The extent of functional redundancy changes as species' roles shift in different environments. Proceedings of the National Academy of Sciences of the United States of America, 112, 14888–14893. 10.1073/pnas.1505587112 26578806 PMC4672811

[ece310409-bib-0016] Galland, T. , Pérez Carmona, C. , Götzenberger, L. , Valencia, E. , & De Bello, F. (2020). Are redundancy indices redundant? An evaluation based on parameterized simulations. Ecological Indicators, 116, 106488. 10.1016/j.ecolind.2020.106488

[ece310409-bib-0017] Gamfeldt, L. , & Hillebrand, H. (2008). Biodiversity effects on aquatic ecosystem functioning – Maturation of a new paradigm. International Review of Hydrobiology, 93, 550–564. 10.1002/iroh.200711022

[ece310409-bib-0018] Gamfeldt, L. , Hillebrand, H. , & Jonsson, P. R. (2008). Multiple functions increase the importance of biodiversity for overall ecosystem functioning. Ecology, 89, 1223–1231. 10.1890/06-2091.1 18543617

[ece310409-bib-0019] Gamfeldt, L. , & Roger, F. (2017). Revisiting the biodiversity–ecosystem multifunctionality relationship. Nature Ecology and Evolution, 1, 168. 10.1038/s41559-017-0168 28812584

[ece310409-bib-0020] Garland, G. , Banerjee, S. , Edlinger, A. , Miranda Oliveira, E. , Herzog, C. , Wittwer, R. , Philippot, L. , Maestre, F. T. , & Heijden, M. G. A. (2021). A closer look at the functions behind ecosystem multifunctionality: A review. Journal of Ecology, 109, 600–613. 10.1111/1365-2745.13511

[ece310409-bib-0021] Hector, A. , & Bagchi, R. (2007). Biodiversity and ecosystem multifunctionality. Nature, 448, 188–190. 10.1038/nature05947 17625564

[ece310409-bib-0022] Hill, M. O. (1973). Diversity and evenness: A unifying notation and its consequences. Ecology, 54, 427–432. 10.2307/1934352

[ece310409-bib-0023] Hillebrand, H. , & Kunze, C. (2020). Meta‐analysis on pulse disturbances reveals differences in functional and compositional recovery across ecosystems. Ecology Letters, 23, 575–585. 10.1111/ele.13457 31943698

[ece310409-bib-0024] Hillebrand, H. , & Matthiessen, B. (2009). Biodiversity in a complex world: Consolidation and progress in functional biodiversity research. Ecology Letters, 12, 1405–1419. 10.1111/j.1461-0248.2009.01388.x 19849711

[ece310409-bib-0025] Jing, X. , Prager, C. M. , Classen, A. T. , Maestre, F. T. , He, J.‐S. , & Sanders, N. J. (2020). Variation in the methods leads to variation in the interpretation of biodiversity–ecosystem multifunctionality relationships. Journal of Plant Ecology, 13, 431–441. 10.1093/jpe/rtaa031

[ece310409-bib-0026] Johnson, K. H. , Vogt, K. A. , Clark, H. J. , Schmitz, O. J. , & Vogt, D. J. (1996). Biodiversity and the productivity and stability of ecosystems. Trends in Ecology & Evolution, 11, 372–377. 10.1016/0169-5347(96)10040-9 21237882

[ece310409-bib-0027] Jones, H. (2013). Plants and microclimate: A quantitative approach to environmental plant physiology (3rd ed.). Cambridge University Press. 10.1017/CBO9780511845727

[ece310409-bib-0028] Kirwan, L. , Connolly, J. , Finn, J. A. , Brophy, C. , Lüscher, A. , Nyfeler, D. , & Sebastià, M.‐T. (2009). Diversity–interaction modeling: Estimating contributions of species identities and interactions to ecosystem function. Ecology, 90, 2032–2038. 10.1890/08-1684.1 19739365

[ece310409-bib-0029] Kremen, C. (2005). Managing ecosystem services: What do we need to know about their ecology? Ecology Letters, 8, 468–479. 10.1111/j.1461-0248.2005.00751.x 21352450

[ece310409-bib-0030] Küpper, H. , Šetlík, I. , Seibert, S. , Prášil, O. , Šetlikova, E. , Strittmatter, M. , Levitan, O. , Lohscheider, J. , Adamska, I. , & Berman‐Frank, I. (2008). Iron limitation in the marine cyanobacterium Trichodesmium reveals new insights into regulation of photosynthesis and nitrogen fixation. The New Phytologist, 179, 784–798. 10.1111/j.1469-8137.2008.02497.x 18513224

[ece310409-bib-0031] Larsen, T. H. , Williams, N. M. , & Kremen, C. (2005). Extinction order and altered community structure rapidly disrupt ecosystem functioning. Ecology Letters, 8, 538–547. 10.1111/j.1461-0248.2005.00749.x 21352458

[ece310409-bib-0032] Lawton, J. H. , & Brown, V. K. (1994). Redundancy in ecosystems. In E.‐D. Schulze & H. A. Mooney (Eds.), Biodiversity and ecosystem function (pp. 255–270). Springer Berlin Heidelberg. 10.1007/978-3-642-58001-7_12

[ece310409-bib-0033] Lefcheck, J. S. , Byrnes, J. E. K. , Isbell, F. , Gamfeldt, L. , Griffin, J. N. , Eisenhauer, N. , Hensel, M. J. S. , Hector, A. , Cardinale, B. J. , & Duffy, J. E. (2015). Biodiversity enhances ecosystem multifunctionality across trophic levels and habitats. Nature Communications, 6, 6936. 10.1038/ncomms7936 PMC4423209 25907115

[ece310409-bib-0034] Li, Y. , Ge, Y. , Wang, J. , Shen, C. , Wang, J. , & Liu, Y. (2021). Functional redundancy and specific taxa modulate the contribution of prokaryotic diversity and composition to multifunctionality. Molecular Ecology, 30, 2915–2930. 10.1111/mec.15935 33905157

[ece310409-bib-0035] Ma, Z. , Liu, H. , Mi, Z. , Zhang, Z. , Wang, Y. , Xu, W. , Jiang, L. , & He, J.‐S. (2017). Climate warming reduces the temporal stability of plant community biomass production. Nature Communications, 8, 15378. 10.1038/ncomms15378 PMC5436222 28488673

[ece310409-bib-0036] Maestre, F. T. , Castillo‐Monroy, A. P. , Bowker, M. A. , & Ochoa‐Hueso, R. (2012). Species richness effects on ecosystem multifunctionality depend on evenness, composition and spatial pattern. Journal of Ecology, 100, 317–330. 10.1111/j.1365-2745.2011.01918.x

[ece310409-bib-0037] Manning, P. , Van Der Plas, F. , Soliveres, S. , Allan, E. , Maestre, F. T. , Mace, G. , Whittingham, M. J. , & Fischer, M. (2018). Redefining ecosystem multifunctionality. Nature Ecology and Evolution, 2, 427–436. 10.1038/s41559-017-0461-7 29453352

[ece310409-bib-0038] Marquard, E. , Weigelt, A. , Roscher, C. , Gubsch, M. , Lipowsky, A. , & Schmid, B. (2009). Positive biodiversity‐productivity relationship due to increased plant density. Journal of Ecology, 97, 696–704. 10.1111/j.1365-2745.2009.01521.x

[ece310409-bib-0039] Marquard, E. , Weigelt, A. , Temperton, V. M. , Roscher, C. , Schumacher, J. , Buchmann, N. , Fischer, M. , Weisser, W. W. , & Schmid, B. (2009). Plant species richness and functional composition drive overyielding in a six‐year grassland experiment. Ecology, 90, 3290–3302. 10.1890/09-0069.1 20120799

[ece310409-bib-0040] Mccauley, D. J. , Gellner, G. , Martinez, N. D. , Williams, R. J. , Sandin, S. A. , Micheli, F. , Mumby, P. J. , & Mccann, K. S. (2018). On the prevalence and dynamics of inverted trophic pyramids and otherwise top‐heavy communities. Ecology Letters, 21, 439–454. 10.1111/ele.12900 29316114

[ece310409-bib-0041] Meyer, S. T. , Ptacnik, R. , Hillebrand, H. , Bessler, H. , Buchmann, N. , Ebeling, A. , Eisenhauer, N. , Engels, C. , Fischer, M. , Halle, S. , Klein, A.‐M. , Oelmann, Y. , Roscher, C. , Rottstock, T. , Scherber, C. , Scheu, S. , Schmid, B. , Schulze, E.‐D. , Temperton, V. M. , … Weisser, W. W. (2018). Biodiversity–multifunctionality relationships depend on identity and number of measured functions. Nature Ecology and Evolution, 2, 44–49. 10.1038/s41559-017-0391-4 29180710

[ece310409-bib-0042] Moi, D. A. , Romero, G. Q. , Antiqueira, P. A. P. , Mormul, R. P. , Teixeira De Mello, F. , & Bonecker, C. C. (2021). Multitrophic richness enhances ecosystem multifunctionality of tropical shallow lakes. Functional Ecology, 35, 942–954. 10.1111/1365-2435.13758

[ece310409-bib-0043] Mori, A. S. , Furukawa, T. , & Sasaki, T. (2013). Response diversity determines the resilience of ecosystems to environmental change. Biological Reviews, 88, 349–364. 10.1111/brv.12004 23217173

[ece310409-bib-0044] Mori, A. S. , Isbell, F. , Fujii, S. , Makoto, K. , Matsuoka, S. , & Osono, T. (2016). Low multifunctional redundancy of soil fungal diversity at multiple scales. Ecology Letters, 19, 249–259. 10.1111/ele.12560 26689733

[ece310409-bib-0045] Mori, A. S. , Isbell, F. , & Seidl, R. (2018). β‐Diversity, community assembly, and ecosystem functioning. Trends in Ecology & Evolution, 33, 549–564. 10.1016/j.tree.2018.04.012 29807839 PMC7612777

[ece310409-bib-0046] Naeem, S. (2008). Species redundancy and ecosystem reliability. Conservation Biology, 12, 39–45. 10.1111/j.1523-1739.1998.96379.x

[ece310409-bib-0047] Pasari, J. R. , Levi, T. , Zavaleta, E. S. , & Tilman, D. (2013). Several scales of biodiversity affect ecosystem multifunctionality. Proceedings of the National Academy of Sciences of the United States of America, 110, 10219–10222. 10.1073/pnas.1220333110 23733963 PMC3690867

[ece310409-bib-0048] Peter, H. , Ylla, I. , Gudasz, C. , Romaní, A. M. , Sabater, S. , & Tranvik, L. J. (2011). Multifunctionality and diversity in bacterial biofilms. PLoS ONE, 6, e23225. 10.1371/journal.pone.0023225 21850263 PMC3151291

[ece310409-bib-0049] Pinheiro, L. F. S. , Pilon, N. A. L. , Rossatto, D. R. , & Kolb, R. M. (2022). Shade drives plant community changes of ground‐layer savanna vegetation: Short‐term changes under an experimental approach. Journal of Vegetation Science, 33, e13118. 10.1111/jvs.13118

[ece310409-bib-0050] Roscher, C. , Schumacher, J. , Gubsch, M. , Lipowsky, A. , Weigelt, A. , Buchmann, N. , Schmid, B. , & Schulze, E.‐D. (2012). Using plant functional traits to explain diversity–productivity relationships. PLoS One, 7, e36760. 10.1371/journal.pone.0036760 22623961 PMC3356333

[ece310409-bib-0051] Slade, E. M. , Kirwan, L. , Bell, T. , Philipson, C. D. , Lewis, O. T. , & Roslin, T. (2017). The importance of species identity and interactions for multifunctionality depends on how ecosystem functions are valued. Ecology, 98, 2626–2639. 10.1002/ecy.1954 28722121

[ece310409-bib-0052] Solan, M. (2004). Extinction and ecosystem function in the marine benthos. Science, 306, 1177–1180. 10.1126/science.1103960 15539601

[ece310409-bib-0053] Soliveres, S. , Van Der Plas, F. , Manning, P. , Prati, D. , Gossner, M. M. , Renner, S. C. , Alt, F. , Arndt, H. , Baumgartner, V. , Binkenstein, J. , Birkhofer, K. , Blaser, S. , Blüthgen, N. , Boch, S. , Böhm, S. , Börschig, C. , Buscot, F. , Diekötter, T. , Heinze, J. , … Allan, E. (2016). Biodiversity at multiple trophic levels is needed for ecosystem multifunctionality. Nature, 536, 456–459. 10.1038/nature19092 27533038

[ece310409-bib-0054] Spehn, E. M. , Hector, A. , Joshi, J. , Scherer‐Lorenzen, M. , Schmid, B. , Bazeley‐White, E. , Beierkuhnlein, C. , Caldeira, M. C. , Diemer, M. , Dimitrakopoulos, P. G. , Finn, J. A. , Freitas, H. , Giller, P. S. , Good, J. , Harris, R. , Högberg, P. , Huss‐Danell, K. , Jumpponen, A. , Koricheva, J. , … Lawton, J. H. (2005). Ecosystem effects of biodiversity manipulations in European grasslands. Ecological Monographs, 75, 37–63. 10.1890/03-4101

[ece310409-bib-0055] Tilman, D. (1999). The ecological consequences of changes in biodiversity: A search for general principles. Ecology, 80, 1455–1474. 10.1890/0012-9658(1999)080[1455:tecoci]2.0.co;2

[ece310409-bib-0056] Van Der Plas, F. , Manning, P. , Allan, E. , Scherer‐Lorenzen, M. , Verheyen, K. , Wirth, C. , Zavala, M. A. , Hector, A. , Ampoorter, E. , Baeten, L. , Barbaro, L. , Bauhus, J. , Benavides, R. , Benneter, A. , Berthold, F. , Bonal, D. , Bouriaud, O. , Bruelheide, H. , Bussotti, F. , … Fischer, M. (2016). Jack‐of‐all‐trades effects drive biodiversity–ecosystem multifunctionality relationships in European forests. Nature Communications, 7, 11109. 10.1038/ncomms11109 PMC4820852 27010076

[ece310409-bib-0057] Walker, B. H. (1992). Biodiversity and ecological redundancy. Conservation Biology, 6, 18–23. 10.2307/2385847

[ece310409-bib-0058] Wang, H. , Lv, G. , Cai, Y. , Zhang, X. , Jiang, L. , & Yang, X. (2021). Determining the effects of biotic and abiotic factors on the ecosystem multifunctionality in a desert‐oasis ecotone. Ecological Indicators, 128, 107830. 10.1016/j.ecolind.2021.107830

[ece310409-bib-0059] Wang, Y. , Liu, B. , Zhao, J. , Ye, C. , Wei, L. , Sun, J. , Chu, C. , & Lee, T. M. (2022). Global patterns and abiotic drivers of ecosystem multifunctionality in dominant natural ecosystems. Environment International, 168, 107480. 10.1016/j.envint.2022.107480 36007300

[ece310409-bib-0060] Yachi, S. , & Loreau, M. (1999). Biodiversity and ecosystem productivity in a fluctuating environment: The insurance hypothesis. Proceedings of the National Academy of Sciences of the United States of America, 96, 1463–1468. 10.1073/pnas.96.4.1463 9990046 PMC15485

[ece310409-bib-0061] Yodzis, P. (1980). The connectance of real ecosystems. Nature, 284, 544–545. 10.1038/284544a0

[ece310409-bib-0062] Zavaleta, E. S. , Pasari, J. R. , Hulvey, K. B. , & Tilman, G. D. (2010). Sustaining multiple ecosystem functions in grassland communities requires higher biodiversity. Proceedings of the National Academy of Sciences of the United States of America, 107, 1443–1446. 10.1073/pnas.0906829107 20080690 PMC2824370

